# How culturally competent are hospitals in Israel?

**DOI:** 10.1186/s13584-018-0255-7

**Published:** 2018-11-19

**Authors:** Michal Schuster, Irit Elroy, Bruce Rosen

**Affiliations:** 1 Unit for Language Facilitation and Empowerment, University of the Free State, POB 339, Bloemfontein, 9300 Republic of South Africa; 2 Myers-JDC-Brookdale Institute, Jerusalem, Israel

**Keywords:** Cultural competence, Language accessibility, Equality, Health policy, Standards

## Abstract

**Background:**

Cultural competence (CC) in health systems is the ability to provide care to patients with different values, beliefs and behaviors, and to match the care to their social, cultural and linguistic needs. In 2011, the Director-General of Israel’s Ministry of Health issued a cultural competence directive to health care providers that sought to minimize health inequalities caused by cultural and linguistic gaps. This study assesses the status of organizational CC in Israeli general hospitals in the wake of the 2011 directive.

**Method:**

Organizational CC was assessed using a 75-item structured questionnaire based on the 2011 directive and on international standards. Data were gathered via interviews conducted between December 2012 and February 2014. 35 of Israel’s 36 general hospitals participated in the study, for a response rate of 97%.

A composite CC score was calculated for each hospital as the average of the 75 items in the questionnaire.

**Results:**

The average composite score of all the hospitals was low to moderate (2.3 on a scale of 0–4), the median score was 2.4, and the range of composite scores was large, 0.7–3.2. The interquartile range was [1.94, 2.57].

Hospital CC is positively associated with non-private ownership status and location in the southern or central districts. Still, these differences are not statistically significant and immutable hospital characteristics such as ownership status and location account for only 21% of the inter-hospital variation in CC. This suggests that hospital leaders have significant discretion in the priority to be given to CC.

Dimensions of CC with relatively low average scores include hospital connections with the community (1.28), staff training on CC (1.35), oral translation (i.e. interpreting) during treatment (1.62), and CC adaptation of human resources recruitment and evaluation (1.64). These areas appear to be particularly in need of improvement.

**Conclusion:**

The study findings suggest that hospitals and policy-makers can take significant steps to improve CC; these include setting more concrete and measurable implementation guidelines. We conclude with suggestions for policy and practices to improve cultural competence in the health system.

**Electronic supplementary material:**

The online version of this article (10.1186/s13584-018-0255-7) contains supplementary material, which is available to authorized users.

## Background

Health organizations and providers serve patients from a variety of religious, cultural and linguistic backgrounds. Those differences in patient characteristics are associated with different needs and different levels of accessibility to health information and services.

The realization that this diversity is both a challenge and an opportunity has led to a shift towards providing services that are tailored to the cultural needs and preferences of the patients. “Cultural Competence” (CC) in health systems refers to the system’s ability to provide care to patients with diverse values, beliefs and needs and adapt the treatment to their social, cultural and linguistic needs and behaviors [2].

Health systems have a variety of motivations and strategies for providing culturally competent services. In some countries, CC is embedded into all services and institutions as a value and an ideology. In others, CC aims to minimize inequality in health caused by cultural differences or serves as a tool to minimize institutionalized racism.

As part of the efforts of Israel’s Ministry of Health (MoH) to minimize inequalities that are attributed to cultural factors, it has adopted the concept of CC in health care, inter alia through a 2011 Director-General’s Directive (DGD) [3, 4].

This paper presents the status of CC in Israeli hospitals - i.e. their ability to provide services that are tailored to diverse cultural needs - as measured using a structured questionnaire. It is a part of a larger study, which also included in-depth interviews of senior hospital managers (which also explored factors contributing to, and hindering, CC) and observations to examine the languages used in signs and other written materials posted in the public space; the main results from those components of the study have been reported elsewhere [5, 6].

## Theoretical background

### Organizational CC

The prominence of CC has been developing over the past decades in Western Countries. The United States, Canada and Australia are states in which comprehensive Cultural Competence in healthcare is implemented. In most European metropolitan areas, health services are required to provide culturally competent care in order to reduce inequality for patients from diverse ethnic backgrounds [7, 8].

Saha et el. describe the evolution of CC in healthcare, starting from the later 1980s. The first groups to enjoy cross-cultural care were immigrants, and the concept later expanded to all minority groups (especially those who were most affected by racial disparities in the quality of care). Another expansion of the concept occurred as CC went beyond the interpersonal domain to the organizational and community domains [9].

CC in healthcare is therefore manifested in three tiers: systemic, organizational and clinical-interpersonal. As the current study evaluated mainly organizational CC, our literature review refers to fundamental policies and studies such as setting policy for treating cultural minorities; appointing a person responsible for CC; providing language assistance and promoting ethnic diversity in employment [2, 10].

In some countries, where multi-culturalism is a core value of society, concepts of CC are embedded in legislation, regulations, and inter-organizational policies. CC values and actions are promoted alongside a larger endeavor that aims to reduce inequity and inequality in health [11].

Two documents that address the systemic and organizational aspects of CC deserve special note. The 14 standards for Culturally and Linguistically Appropriate Services (CLAS), issued by the US Department of Health and Human Services [1, 12] has affected practice in many health systems worldwide. The second document is the Amsterdam Declaration on Migrant Friendly Hospitals in Europe, which has been endorsed by many European and international institutions [13].

### Evaluation of organizational CC

Obtaining information about organizational CC is critical to determining whether clients are receiving culturally-competent care from all facets of an organization [14]. Paez and colleagues found that CC in community clinics was positively related to interpersonal CC skills [15]. Most studies of organizational CC concentrate on language barriers, the need to train providers to care for diverse patient populations [16] and health-education programs for targeted communities [17]. Fewer studies focus on strategic planning [18] and administrative aspects of organizational CC, but they do make a link between CC and quality [19], improved financial outcomes through cost savings, increased market share, and improved efficiency [19]. Only a handful of studies have made a direct link between CC and the reduction of racial/ethnic disparities in health care [20, 21].

Studies examining organizational aspects of CC have used interviews with CEOs [20] surveys and focus groups [16] and a systemic review of healthcare interventions in community clinics [21]. In another study, the authors designed a mapping tool of 53 measure for organizational CC and examined whether each of them was met, either fully or partially [14]. Weech-Maldonado and colleagues developed a designated tool, CCATH, that was aimed to measure the implementation of the CLAS standards in 135 California hospitals [22]. Their findings suggest, similar to previous studies in other US states, that the hospitals perform relatively well on specific patient-related measure, but less well on measures related to management commitment, and integration of CC into organizational systems (e.g. HR or information systems) [22].

In summary, most studies to date have evaluated CC-related personal skills and there are significantly less studies on organizational CC. Apart from a few countrywide-American studies [20, 22], no studies have yet measured it on a national level, as the current study does.

### CC in Israeli healthcare

Israel has a national health insurance system with universal coverage. All citizens can choose from among four competing health plans, which are responsible for ensuring that their members receive all necessary health services consistent with a government-determined benefits package.

The vast majority of the hospitals are public – i.e. operated by the MoH, non-for-profit organizations or the two largest health plans (HMOs). Most hospital services are purchased by the health plans.

Ownership of acute care hospital beds is divided as follows: Government/MoH – 47%, Clalit (the largest health plan) – 29%, other non-profits – 21%, for profits – 3%. The Ministry of Health regulates all the hospitals, but naturally it has more direct influence on those hospitals that it owns and operates. Interestingly, all of the hospitals in Jerusalem (Israel’s capital) are independent non-profits, which are owned by neither the government nor a health plan.

The population in Israel is very heterogeneous. In 2018, Israel was populated by 8.8 million people. 75% are Jews, 21% are Muslim, Arab-Christian or Druze and about 5% are other Christians, other religions or without religious affiliation [23]. [The first official language is Hebrew, spoken by the majority of the citizens as first or second language. The second official language is Arabic (spoken by about 21% as a first language. Relatively high concentrations of Arabs can be found in Jerusalem, the Northern district and the Southern district.

The de-facto second language is English, taught from first grade (or sometimes before) and appears in most public spaces and scenes – media, commerce, business and academia. As an immigration state since its establishment, about 37 languages and dialects are spoken (or signed, referring to Israeli Sign Language) [24].

The diversity of backgrounds inevitably creates challenges to accessibility to, and the proper consumption of, health services [4]. Despite the universal insurance coverage in Israel, there are significant health and health care disparities across regions, socio-economic groups and ethnic groups. There are also a wide range of governmental and non-governmental efforts underway to reduce these disparities [25].

Local initiatives to provide culturally-competent care have existed in Israel since the 1990s. There have been efforts to promote CC at the organizational level, mainly through non-governmental organizations such as the Jerusalem Intercultural Center, which has promoted and facilitated CC in healthcare organizations through staff training, consulting on language accessibility issues etc. [26, 27]. However, nation-wide recognition and regulation was formalized only in 2011, when the Director General of the Ministry of Health published a special directive that aims to minimize health inequalities caused by cultural and linguistic gaps, create standards for CC in health organizations and improve the accessibility of health services to all populations [3, 22, 28]. Some of the guidelines (e.g. most sections that refer to language accessibility) are mandatory, and some are phrased as recommendations or general guidelines. The seven main sections focus on the following areas: developing organizational infrastructure to provide cultural and linguistically competent care; translation and interpreting; training of the staff members, in all professions, in cultural competence; developing the physical infrastructure (e.g. multilingual signs); maximal adaptation of the services to the specific populations of each hospital or clinic, according to linguistic and cultural data that will be collected [reference]. Organizations were given 2 years to implement the directive, and the guidelines are now being monitored/reviewed by the Ministry of Health.

The current study is the first to measure organizational aspects of CC in Israel.

## Methods

### Study hospitals

At the beginning of 2012, the Ministry of Health listed 45 general hospitals as being in operation. We excluded from our study three hospitals because they treated only obstetrics patients and one hospital because it closed prior to the study’s implementation. In addition, the two hospitals which compose Beilinson Medical Center were treated as a single unit, as were the four hospitals operating under the Assuta framework and the two hospitals operating under the Elisha framework.

Accordingly, the study sample consisted of 36 “hospital units”, henceforth referred to simply as “hospitals”. Thirty five(35) of the 36 agreed to participate and one refused, so that the response rate was 97%.Of the participating hospitals, 11 are government hospitals, 20 are non-profits and five are private.

### Research tools and methodology

To measure organizational CC in hospitals,^1^ we created a structured questionnaire for mapping CC, where the dimensions of CC measured by the questionnaire are based on the DGD and on international standards.^2^ The tool (Additional file 1) contains 75 statements grouped into 10 broader topics^3^:

1. Organizational policy on CC care
2. Appointment and development of a CC coordinator
3. Oral translation (i.e. interpreting) during treatment
4. Translation of official forms and written medical material
5. Religious and cultural services
6. Adaptation of the physical environment to the patient population
7. Staff training on CC
8. Hospital contacts with the community
9. Cultural adaptation of human resources (recruitment and evaluation)
10. Data Collection and management of patient diversity.

The interviews were conducted either by telephone or face to face and they took place between December 2012 and February 2014. We interviewed either the CC Coordinator or a senior administrative staff member with organizational responsibility for CC. They graded the extent that each of 75 statements was applicable to their hospital on a 5-point scale, as follows:

·0 - Non-existent and not planned;
·1 - Planned for the coming year;
·2 - True to a small extent/in preliminary implementation stages;
·3 - True to a moderate extent/partially implemented;
·4 - True to a large extent/fully implemented.

Additional responses coded were: “not applicable” and “don’t know”.

### The CC composite score (CCCS)

To measure CC we created an index: a score that is comprised of the average score obtained in the 75 statements in our structured questionnaire. Each of the 75 statements received an equal weight in the sum-total grade. The average score in the CC composite score (CCCS) reflects the hospital’s overall level of organizational CC.

## Results

The average CC composite score (CCCS) for the 35 general hospitals was 2.30 and the median was 2.38^4^. The average scores hospitals received on the index ranged between 0.7–3.2. The average score was between 2 and 3 for 23 hospitals, above 3 for 2 hospitals and below 2 for 10 hospitals. The interquartile range was [1.94, 2.57].

We examined the relation between the CC composite score (CCCS) for each hospital and their/its key characteristics. We also examined the relation between the CCCS and specific organizational measures taken by the hospitals.

### Relation between the hospital’s characteristics and the CC level

We examined a possible relation between the hospitals’ CCCS and each of the following five hospital characteristics: organizational affiliation, status in the accreditation process, district, size (less or more than 400 beds),^5^ and geographical centrality (using the CBS 5-point peripherality index).

As can be seen in Table 1, several characteristics were associated with the hospital’s CC level, as described below:

**Table 1:** Cultural Competence Composite Scores, by Hospital Characteristics

Variable	N	Median CCCS	Average CCCS	Significance level
ALL HOSPITALS	35	2.37	2.24	NA
Organizational Affiliation				
Government / Public	30	2.38	2.30	
Private	5	2.20	1.86	0.04
Accreditation Process				
Completed or initiated	10	2.40	2.32	
No involvement	25	2.29	2.03	.00
District				
South or Center	16	2.44	2.36	
North or Jerusalem	19	2.32	2.13	0.40
Peripherality Index				
Center (1 to 3)	18	2.39	2.26	
Periphery (4 or 5)	17	2.37	2.21	0.95
Hospital Size				
Medium-Large (400+ beds)	17	2.41	2.28	
Small (under 400 beds)	18	2.37	2.20	0.22
Sector				
General	29	2.41	2.26	
Arab	6	2.31	2.16	0.95

*Organizational affiliation* had the greatest relation to the CC status of hospitals. Government and public hospitals (which had similar CC levels) were found to be more culturally competent than private ones, with a gap of almost one-half point in the 5-point composite score. The difference was statistically significant at the 0.05 level.

*The JCI*^6^ *accreditation process:* hospitals that had completed, or initiated, the process of receiving a standards seal received a higher CCCS than those that have not begun the accreditation process, with a gap of approximately one-third of a point in the composite score. The difference was statistically significant at the 0.01 level.

*Geographical district:* hospitals in the southern or central districts received a higher CCCS than those in the north or in Jerusalem (whether taken together or separately), with a gap of about a quarter of a point in the composite score. The difference was not statistically significant.

An OLS regression analysis was carried out to assess the independent effect of each of the hospital characteristics on the CCCS and to assess the extent to which, taken together, they account for the variance in the CCCS. As indicated in Table 2, none of the hospital characteristics had a statistically significant independent effect on CCCS at the 0.05 level, and only the ownership variable was significant at the 0.10 level. The R-squared was 0.21.

**Table 2:** Regression of cultural competence scores (CCCS) on hospital characteristics

Characteristic	Coefficient	Standard error	T statistic	Significance
Constant	2.20	0.31	6.95	.00
Private ownership	−0.66	0..35	−1.89	.07
Initiated accreditation process	0.14	0.25	0.55	.59
South or center	0.36	0.23	1.58	.13
Periphery	0.02	0.21	0.10	.92
Medium-Large	−0.25	0.25	−0.99	.33
Arab hospital	−0.12	0.32	−0.39	.70

R-squared = 0.21

Appendix 1 presents data on average CCCS by respondent profession and seniority. Overall, average CCCS varied only slightly among these groups and none of the differences were statistically significant.

### Analysis of CC, by topic and statement

To learn if certain aspects of CC were implemented more than others, we compared the average scores for each of the 10 topics. Table 3 presents the mean score across all 35 hospitals (on a 0–4 scale), listed from highest to lowest:

**Table 3:** Scores on the CC Index, by Topic, for all 35 hospitals

Topic	Mean Score on CC Index
1. Religious and cultural services in the hospital	3.10
2.Translation of official forms and written medical material	2.79
3. Adaptation of the physical environment to patient population	2.48
4. Organizational policy of hospital on CC care	2.43
5. Data collection and management on patient diversity	2.00
6. Appointment and development of CC coordinator	1.77
7. Cultural adaptation of Human Resources recruitment and evaluation	1.64
8. Oral translation (i.e. interpreting) during treatment	1.62
9. Staff training on CC	1.35
10. Hospital connections with the community	1.28

Religious and cultural services received the highest score of all 10 topics. Relatively high scores were also received for the following topics: translation of official forms, adaptation of the physical environment, and organizational policy. Possible reasons are that they are mandatory, they have relatively clear DGD guidelines and failure to provide them could result in legal action. In contrast, the implementation of the four topics that received low scores demands financial resources and cross-organizational cooperation. They are considered recommendations, which may also contribute to their low level of implementation. It is noteworthy that these findings are non-congruent with the findings of Weech-Maldonado (2010) and similar studies in other U.S stated. The American studies found that hospitals had better performance in patient-related cultural competency measures (e.g. data collection on, interpreter services, and clinical cultural competency practices) compared their underperformance in the subdomains of leadership and strategic planning, and community representation, suggesting that the hospitals leg behind in integrating CC into organizational and management practices. A possible explanation to the differences found, is that the organizational steps in the Israeli directive were mandatory, a fact that promoted their implementation.

### Analysis according to the obligatory status of the guideline

The average score on statements representing mandatory DGD guidelines (i.e. guidelines phrased as “all hospitals must/are required to…”) was higher (2.36) than the average on statements reflecting recommended guidelines (i.e. “it is recommended that…”) (2.12).

### Relationship between CCCS and key organizational measures taken by the hospitals

We examined the relationships between the hospitals’ CCCS and key DGD-mandated measures: appointing a CC coordinator, the existence of a steering committee and a written work plan. These measures are described in the literature as essential in promoting CC in hospitals.

As noted above, 46% of the hospitals had a CC coordinator; 69% had appointed a steering committee (but only in 60% did it meet regularly), and 74% had a written work plan.

Hospitals that implemented these measures, received higher scores in the CC composite score than those that did not (Table 4). Still, the cross-sectional nature of the study precludes causal conclusions. Moreover, a multivariate regression of the index on these specific measures is problematic because the measures are themselves components of the index. Accordingly, all we can say is that the bivariate findings suggest that formulating a work plan and appointing of a steering committee may have a marked impact on the CCCS and this is less likely to be the case for the appointment of a CC coordinator.

**Table 4:** Cultural competence composite scores according to CC organizational measures taken by hospitals

Variable	Cultural competence composite score
Appointing a CC coordinator	
Yes	2.46
No	2.05
Appointing CC Steering committee	
Yes	2.42
No	1.85
Steering committee meetings	
Regular	2.41
Irregular	1.98
Formulating a work plan	
Yes	2.42
No	1.71

## Discussion

The study found that the average CCCS of all Israeli hospitals was low to moderate (2.3 on a scale of 0–4), indicating that there is substantial need for improvement. The study also found that the range of scores was large, 0.7–3.2, suggesting that improvement is possible within the Israeli environment, and that there are lessons to be learned from the hospitals with the highest CC scores.

In an attempt to understand what impacts hospital levels of CC, we hypothesized a relation between CC and a number of organizational characteristics, as well as to some CC-promoting actions. Indeed, government and public hospitals were on average more culturally competent than private ones (albeit the difference was not statistically significant). A possible explanation is that in Israel, the Ministry of Health, which is both a regulator and a service provider, manages many public hospitals itself. Thus, the MoH has more communication with, and influence over hospitals that it owns and therefore can encourage and monitor the implementation of the Directive. The ownership-based difference was also noted in Weech-Maldonado et al., who explained that in the difference in missions and markets of both types. They further noted research is needed on the business case for cultural competency, showing how organizational cultural competency activities may relate to patient satisfaction, revenues, and ultimately financial performance.

In addition, in the bivariate analysis we found that the accreditation process (in the framework of JCI) is associated with a somewhat higher level of CC in hospitals; preparing the accreditation survey, which examines patient safety, including aspects that relate directly to clear communication and accessibility of services, may contribute to greater implementation of CC-related DGD-mandated measures.^7^ However, the multivariate analysis revealed that the relationship between JCI accreditation and CCCS is not a strong, independent relationship. This may be because the JCI process deals with only a limited part of CC – language accessibility.

However, a multivariate analysis found that none of the immutable hospital characteristics measured in the study had a significant, independent effect on overall CC at the 0.05 level, and only ownership and location had a significant effect on the 0.10 level. Moreover, the hospital characteristics, taken together, account for only 21% of the inter-hospital variation in CC. This suggests that hospital leaders have significant discretion in the priority to be given to CC; the CC level of their hospitals is far from determined by immutable hospital characteristics.

Interestingly, average CC was relatively low for the Jerusalem region. All of the Jerusalem hospitals are independent non-profits. Moreover, several of them are located in East Jerusalem and serve exclusively the Arab population. The East Jerusalem hospitals are also relatively small, have limited resources, and because of the political situation they are less closely regulated by the Ministry of Health. Two of the three East Jerusalem hospitals had quite low average CCCS scores, and this brought down the average for Jerusalem as a whole. Their low scores may be related to the fact that they serve a relatively homogenous population (i.e. Arabs), while other factors may be their size, their limited resources, and the limited government regulation due to political sensitivities.

A related point is that the CCCS is slightly below average for hospitals predominantly serving the Arab sector. The relatively low scores for some of the East Jerusalem hospitals are offset by scores close to the average for the Nazareth hospitals.

The study also examined the relationship between specific organizational measures and overall CC. While the nature of the data set preclude causal analysis, the associations identified do suggest that formulating a work plan and appointing a steering committee may have a sizable effect on the CC composite score, and that this effect is likely greater than the effect of the appointment of a CC coordinator. If this is indeed the case, a possible explanation would be that work plans and steering committees involve more people in promoting the CC agenda than does appointing an individual to serve as a CC coordinator.

The actions taken in hospitals are related primarily to DGD mandatory guidelines. Three possible explanations for this are as follows: 1) the mandatory guidelines are fairly basic requirements of CC services; 2) some of the requirements were implemented before the DGD was issued, to overcome substantial, mainly language, barriers with patients; 3) the scarcity of resources caused hospitals to implement mainly the mandatory requirements.

Analysis of the scores and interviewees’ comments (interviewees explained the score they gave on some of the statements) shows that CC is mainly perceived as “language accessibility” (i.e. meeting the linguistic needs of patients) and less as broader adaptations for diverse patients. This higher sensitivity to linguistic issues can be attributed to the clear and practical guidelines for this topic, the daily experience of care-providers who have to overcome language barriers, and to fear of malpractice lawsuits. However, while filling in the questionnaire, the interviewees commented that the financial costs pose a significant obstacle to promoting even the mandatory steps - i.e. signage and translation of forms (for an extended discussion on the promoting and hindering factors of the CC process, see ([5], pp., 16-20)).

Thus, despite the awareness of the importance of CC and the willingness to promote it, the level of CC in Israel’s general hospitals is low to moderate. The guidelines whose implementation was more advanced were the mandatory guidelines. Guidelines that that were concrete and did not require extra budgets - such as appointing a steering committee and writing a work plan - were associated with a higher overall CC level.

## Conclusions

Our study aimed to understand what promotes CC, a policy issued by the Israeli Ministry of Health in 2011 (to be implemented within 2 years). The average cultural competence score of the Israeli public hospitals is 2.24 on a scale of 0–4. This is a low to medium average on the scale. A comparison between the total scores of the hospitals according to the cultural competence index shows that the characteristics with the greatest correlation to the level the cultural competence of the hospitals are organizational affiliation, progress on the JCI accreditation process, and the geographical district. The hospitals receiving the higher scores in the CC index are those which reported having a work plan on cultural competence, as well as a steering committee.

Analysis of the data collected in this tool (as well as other tools used in the larger study) shows that CC is mainly perceived as “language accessibility” (i.e. providing the linguistic needs of patients) and less as broader cultural adaptations. The higher awareness of linguistic issues may be attributed to the clear and practical guidelines for this topic, the daily experience of care providers who have to communicate with language minorities, and to fear of malpractice lawsuits. However, the financial issue poses a significant burden to promoting programs even with regard to language accessibility - such as signage, translation of forms, training of medical interpreters etc.)

Despite the awareness to the importance of CC and the willingness to promote it, the subject is competing with other relevant issues on the agenda of hospital leaders, and in practice, implementation is low to middle. It is important to remember that the study describes the status of CC upon the entry into force of the circular, i.e. in relatively early stages of the process.

Since the score, as we discovered, is not influenced so much from immutable hospital characteristics (e.g. size), it means that hospitals can actively improve their level of CC, by performing the actions that were identified in the study as promoting CC. In order to do so, hospitals need guidance and mentoring for the process in the shape of the JCI accreditation, including clear guidelines or instructions, guidelines such as the directive guidelines on language accessibility or the mandatory guideline to prepare a work-plan.

It should be noted that in the years that followed the issuance of the CC directive, the Ministry of Health has been creating national infrastructure to support hospitals in promoting cultural competence: a medical telephone interpreting service, a training kit on cultural competence for the hospital staff and rewards on such trainings. Following the study, we partnered with the MoH, to improve and upgrade the tool, in order for the Ministry to use it as an assessment tool for Israeli hospitals.

### Limitations

The analysis in the study is based mainly on the self-reporting of hospital office-holders, which could result in subjective reporting and different grading criteria by the interviewers. To help surmount this limitation, we attempted to create uniform grading for the interviewers at the various hospitals by relying on the free wording of interviewees and the descriptions given by interviewers; In addition, to the extent possible, the data from interviewees were cross-referenced with data from other sources: whether supplementary interviews, in-depth interviews with the same interviewees or observations of the linguistic landscape.

In almost all the participating hospitals, the person interviewed for this study was the CC coordinator for the hospital^8^. It is quite possible that being in this position may have led some of them to overstate the extent of CC in their hospital (either consciously or unconsciously). This may have been mitigated, at least to some extent, by the study team’s statement to the respondent that the study would replicated in the future and attention would be focused on the change. Another step undertaken by the study team to reduce the risk of social desirability bias, was to ask respondents to give examples in situations where initial responses roused questions as to their reliability. However, despite these steps, it is quite possible that some element of social desirability remained.

Thus, it seems that the unimpressive CC average scores found in this study are, if anything, an upper bound for the true CC level in Israel in 2012. This only strengthens our conclusion that the situation at that time was unsatisfactory and that measures were needed to improve it.

It is noteworthy that the data collection lasted 1 year; that is, we may have reached hospitals at different stages of progress in the CC process. It is our opinion that a time gap is not essential in the process of cultural accessibility since the CC process is long-term.

Another study limitation is that it was carried out in 2012–2014, and the cultural competence situation may well have changed since then. The hospitals have had more time to understand the CC directive, the MOH has provided technical support, and more hospitals have progressed through the JCI accreditation process. Therefore, the data in this study serves as baseline for future evaluation of CC in general hospitals, since it measures CC status in the beginning of the process, shortly after the dissemination of the DGD. We hope to revisit the situation in the coming years.

## Policy implications

Our study suggests several steps to promote CC in the health system:

Given the difficulty of hospitals in translating some of the DGD guidelines into practice, and the fact that guidelines with clearer instructions received higher scores in our index, it seems important to publish appendices with clear definitions and guidelines for the implementation of standards.

In view of the results that connect between a hospital’s CCCS and the preparation of an annual work plan and the appointment of a steering committee, it seems that implementing these steps is necessary to promote the entire process of CC.

The fact that interviewees kept referring to the lack of resources as a major obstacle, it is important to allocate funds to support CC, e.g. for staff training, a job slot for the CC coordinator and language services.

The scarcity of resources noted by the interviewees also suggests that resource pooling of multiple organizations would increase system efficiency in areas where hospitals have similar needs. The MoH, the HMOs and some NGOs have already started creating such shared resources - e.g. a telephone interpreting service, medical interpreting trainings, and a CC training kit.

We support the MoH’s requirement that the implementation of the Directive should be measured ([3], P. 7). Measuring hospitals by structured standards of quality, and making their licensing conditional, could help promote the topic. In 2016, following the publication of the research report [5] and upon the request of the MoH, the authors created an extensive monitoring tool (based on the mapping tool), that will periodically measure the various aspects of CC in the hospitals.

In the intercultural meeting between professionals and recipients of care, awareness should be raised of the needs of every patient, whether a member of a minority or the majority. It is also important to raise awareness of the needs of patients with physical or cognitive disabilities.

Lastly, it will be important to re-evaluate the hospitals’ CC in the future, to examine the progress made and the extent to which the DGD has been incorporated.

### Additional file

Additional file 1: Cultural Competency (CC) Evaluation Tool. (DOCX 156 kb)

## Appendix 1

**Table 5:** Professional backgrounds and seniority levels of the respondents

Professional background	N	Percent	Average CCCS
Marketing	8	23	2.28
Nursing	16	46	2.34
Management	11	31	2.06
Seniority			
Not senior	9	26	2.26
Senior	26	74	2.23

## Appendix 2

**Table 6:** The ten sub-scales – composition and reliability scores

Scale #	Scale name	Cronbach’s alpha	# of items
1	Organizational policy on CC care	.82	7
2	Appointment and development of a CC coordinator	.77	5
3	Oral translation (i.e. interpreting) during treatment	.94	14
4	Translation of official forms and written medical material	.58	24
5	Religious and cultural services	.45	3
6	Adaptation of the physical environment to the patient population	.80	9
7	Staff training on CC	.59	4
8	Hospital contacts with the community	.66	3
9	Cultural adaptation of human resources (recruitment and evaluation)	.33	2
10	Data Collection and management of patient diversity.	.64	3 s

## Abbreviations

CC: Cultural Competence
CCCS: Cultural Competence Composite Score
CLAS (standards): Culturally and Linguistically Competent Services (see reference [1])
DGD: Director General’s Directive (directive no. 07/11, State of Israel, Ministry of Health. Directive regarding the Cultural and Linguistic Adaptation and improving of access to health care)
HMO: Health Maintenance Fund
JCI: Joint Commission International (see note 4)
MoH: Ministry of Health
NGO: Non-Governmental Organization

## Footnotes

1 The research also included in-depth interviews and structured field observations on cultural adaptation, and findings from those components of the study are reported elsewhere [5, 6]. This paper discusses the findings from the structured questionnaire.

2 The study team considered using the Cultural Competency Assessment Tool for Hospitals (CCATH) that was developed by Weech-Maldonado et al. (2012). The team decided not to use that tool as it was designed for health systems at a more advanced stage of cultural competence implementation. It would not have been appropriate for the Israeli health system in 2012, when it was just beginning to take cultural competence seriously. Some of the items in that tool would not even have been understood by Israeli respondents in 2012 and the tool would not have differentiated well among Israeli hospitals. Having said that, we note that both the CCATH and the tool we developed make serious use of the CLAS standards.

3 Table 3 displays the topics that comprised the various scales, while Appendix 2 indicates a reliability measure for each scale. Cronbach’s alpha was over 0.75 for 4 of the scales, between 0.50 and 0.75 for 4 of the scales and between 0.33 and 0.50 for two of the scales. Not surprisingly, the scales with the lowest reliability scores (human resources CC and religious services CC) were scales with very few items. We retained those two as scales despite their low reliability scores because conceptually the items clearly address related issues.

4 As the study’s unit of analysis is the hospital (and not the patient or population), all findings presented in the main text give equal weight to all participating hospitals. As a sensitivity analysis, we examined how the findings would have been affected by weighting the hospitals by size (using bed count as the size indicator). The weighting had only a minor effect on the key results. For example, the average CCCS increased from 2.24 to 2.38. Moreover, it had only minor effects on the coefficients of the regression

5 It is worth noting that the relationship between CC and hospital size is somewhat complex. For the analyses presented below, the sample was split into two approximately equal-sized groups (Medium-Large for those over 400 beds and Small for those up to 400 beds). In those analyses, almost no bivariate difference was found in average CC level for the two groups, and the size variable was also not significant in the multivariate analysis.

6 JCI – Joint Commission International is a U.S. non-profit organization that accredits and issues a hallmark to health organizations worldwide, focusing on topics of patient safety.

7 The impact of CC on patient outcomes, which is a main goal the JCI accreditation process, was not addressed in the current study. It should be a high priority for future research.

8 In several hospitals, the study team interviewed more than one hospital manager. Even in those hospitals, it was the interview with the CC coordinator that was used in the study reported here. The other interviews contributed to related studies reported elsewhere.
