# Supplementary material for: How culturally competent are hospitals in Israel?
Source: Isr J Health Policy Res. 2018 Nov 19;7:61. doi: 10.1186/s13584-018-0255-7 (PMC6241030; doi:10.1186/s13584-018-0255-7)
Supplement: Supplementary file 1 — Cultural Competency (CC) Evaluation Tool. (DOCX 156 kb) [file 13584_2018_255_MOESM1_ESM.docx]

**Cultural Competency (CC) Evaluation Tool**

| Interviewer’s Name: |  |
| --- | --- |
| Interviewee’s Name: |  |
| Date: |  |
| Position: |  |
| Hospital: |  |
| Organizational Affiliation: |  |

To what extent are the following assertions true about the organization in which you work?

| **Clause** | **Item** | | | | **Not available and not planned** | **Not available and planned for next year** | **In early implementation stages** | **Partially available** | **Fully**  **available** | **Comments** |
| --- | --- | --- | --- | --- | --- | --- | --- | --- | --- | --- |
|  |  |  |  |  |  |  | **True to a small extent** | **True to a moderate extent** | **True to a large extent** |  |
| **Organizational policy on culturally-adapted care** | The question of Cultural Competency (CC) is on the agenda of the hospital management | | | |  |  |  |  |  |  |
|  | The organization has a written policy on the question of CC | | | |  |  |  |  |  |  |
|  | A work plan has been writtn to implement the principles of CC, including priorities, responsible parties and schedules | | | |  |  |  |  |  |  |
|  | An interdisciplinary, multicultural CC steering committee has been appointed | | | |  |  |  |  |  |  |
|  | At what regular intervals does the steering committee meet? | | | |  |  |  |  |  |  |
|  | Resources (funds, time, manpower) are allocated for action in the realm of CC | | | |  |  |  |  |  |  |
|  | Action is being taken to raise awareness of the importance of cultural adaptation in service and care | | | |  |  |  |  |  |  |
| **Appointment and professional development of person in charge of CC** | The person in charge has a **formal** letter of appointment | | | |  |  |  |  |  |  |
|  | Steps have been taken to advise the **healthcare staff** of the existence of a responsible party in charge of CC | | | |  |  |  |  |  |  |
|  | The position of the person in charge of CC has a **prescribed** number of working hours. | | | |  |  |  |  |  |  |
|  | The person in charge of CC is having professional training. Of what sort? | | | |  |  |  |  |  |  |
|  | The person in charge has a **written** work plan | | | |  |  |  |  |  |  |
| **Oral interpretation** **during care** | Staff people who serve as face-to-face interpreters and have had basic **training in medical translation** | | | Hebrew |  |  |  |  |  |  |
|  |  |  |  | English |  |  |  |  |  |  |
|  |  |  |  | Russian |  |  |  |  |  |  |
|  |  |  |  | Arabic |  |  |  |  |  |  |
|  |  |  |  | Amharic |  |  |  |  |  |  |
|  | There is a **list of personnel** trained in medical translation | | | |  |  |  |  |  |  |
|  | **Staff know** of the availability of interpreters at the hospital | | | |  |  |  |  |  |  |
|  | During receipt of service and/or care, **patients** are **offered face-to-face interpretation** of professional translator or personnel trained in translation in a specific language: | | | Hebrew |  |  |  |  |  |  |
|  |  |  |  | English |  |  |  |  |  |  |
|  |  |  |  | Russian |  |  |  |  |  |  |
|  |  |  |  | Arabic |  |  |  |  |  |  |
|  |  |  |  | Amharic |  |  |  |  |  |  |
|  | A translation service is **available** via a telephone hotline | | | |  |  |  |  |  |  |
|  | The **staff were trained** on the use of the translation service via the telephone hotline | | | |  |  |  |  |  |  |
|  | The telephone translation service is **offered** to patients as needed | | | |  |  |  |  |  |  |
| **Translation of official forms and written medical material** | There is a document listing patient rights in the following languages: | Hebrew | | |  |  |  |  |  |  |
|  |  | English | | |  |  |  |  |  |  |
|  |  | Russian | | |  |  |  |  |  |  |
|  |  | Arabic | | |  |  |  |  |  |  |
|  |  | As per the linguistic composition of the main patient groups | | |  |  |  |  |  |  |
|  | Every form to be signed by a patient (informed consent, hospitalization, financial binder) is translated into the following languages: | Hebrew | | |  |  |  |  |  |  |
|  |  | English | | |  |  |  |  |  |  |
|  |  | Russian | | |  |  |  |  |  |  |
|  |  | Arabic | | |  |  |  |  |  |  |
|  |  | As per the linguistic composition of the main patient groups | | |  |  |  |  |  |  |
|  | The procedure of obtaining informed consent is adapted to the patient linguistically and culturally | | | |  |  |  |  |  |  |
|  | Written medical information (e.g., discharge forms, guidelines for further care and promoting health) is translated into the following languages: | Hebrew | | |  |  |  |  |  |  |
|  |  | English | | |  |  |  |  |  |  |
|  |  | Russian | | |  |  |  |  |  |  |
|  |  | Arabic | | |  |  |  |  |  |  |
|  |  | As per the linguistic composition of the main patient groups | | |  |  |  |  |  |  |
|  | Written administrative information (visiting hours, ways of payment etc.) is translated into the following languages: | Hebrew | | |  |  |  |  |  |  |
|  |  | English | | |  |  |  |  |  |  |
|  |  | Russian | | |  |  |  |  |  |  |
|  |  | Arabic | | |  |  |  |  |  |  |
|  |  | As per the linguistic composition of the main patient groups | | |  |  |  |  |  |  |
|  | The official materials were translated by **professional** translators | | | |  |  |  |  |  |  |
|  | The written material suits the **literacy level** of the patients | | | |  |  |  |  |  |  |
|  | The translated material is regularly given to clients who need it | | | |  |  |  |  |  |  |
| **Religious and cultural services** | There are areas for prayer for the main target populations | | | |  |  |  |  |  |  |
|  | In the wards, there is accommodation for holidays and other special days of the main target populations | | | |  |  |  |  |  |  |
|  | During receipt of hospital service and care, a patient’s religious observances are taken into account (e.g., fast days) | | | |  |  |  |  |  |  |
| **Adapting the physical surroundings to the main target populations of the hospital** | The wards/clinics/admission offices are organized in a manner adapted to the hospital’s target populations | | | |  |  |  |  |  |  |
|  | The substance (pictures, content on screens) presented in hospitals is adapted to the main target populations | | | |  |  |  |  |  |  |
|  | The unit dealing with public queries provides a telephone response **within 24 hours** in the different languages: Hebrew, Arabic, Russian and Amharic | | | |  |  |  |  |  |  |
|  | The telephone hotline (to make appointments, receive medical information and information on rights) provides a telephone response in the following languages: Hebrew, Arabic, Russian, Amharic and English within 24 hours | | | |  |  |  |  |  |  |
|  | Signs in Hebrew, Arabic and English and in the languages of the main target populations of the hospital | | Signposting | |  |  |  |  |  |  |
|  |  |  | Information signs (working hours, hospital services available etc.) | |  |  |  |  |  |  |
|  |  |  | Warning signs (isolation room, danger of slipping etc.) | |  |  |  |  |  |  |
|  |  |  | Signs at the entrance to rooms | |  |  |  |  |  |  |
|  |  |  | Emergency guidelines | |  |  |  |  |  |  |
| **Training staff in CC** | **All staff members** had **at least one** training session in the past year on CC | | | |  |  |  |  |  |  |
|  | Intercultural issues are discussed **regularly** in different forums, e.g.: lectures, staff meetings | | | |  |  |  |  |  |  |
|  | Process of absorbing new employees includes training in the subject of CC | | | |  |  |  |  |  |  |
|  | There is **systematic** evaluation of the outcomes of training and its impact on care/health measures | | | |  |  |  |  |  |  |
| **Contact with the community** | Surveys on client satisfaction ask questions about cultural aspects of service and care | | | |  |  |  |  |  |  |
|  | There are procedures for handling crises requiring cultural mediation | | | |  |  |  |  |  |  |
|  | The organization holds culturally-adapted activities in its vicinity to promote community health | | | |  |  |  |  |  |  |
| **Manpower** | In recruiting employees, the cultural profile of the patient population is taken into account | | | |  |  |  |  |  |  |
|  | In the evaluation of employees, aspects of CC are taken into account | | | |  |  |  |  |  |  |
| **Knowledge management** | In the process of registering patients, information is collected on language, origin and cultural preferences of the patients (kashrut, involvement of a religious figure, gender-sensitive care) | | | |  |  |  |  |  |  |
|  | The demographic information collected on patients is analyzed and used in the planning of services and actions to promote health and prevention in these populations | | | |  |  |  |  |  |  |
|  | The staff has access to information on intercultural topics relating to the population of patients (e.g., characteristics of health and illness of the main patient groups, cultural and religious customs etc.) | | | |  |  |  |  |  |  |
| **Additional comments** |  | | | | | | | | | |
